# RE: Universal SARS-CoV-2 testing on admission to the labor and delivery unit: Low prevalence among asymptomatic obstetric patients

**DOI:** 10.1017/ice.2020.382

**Published:** 2020-07-30

**Authors:** Sean Cronin, Megan Piacquadio, Katelyn Brendel, Aden Goldberg, Marco Goldberg, Chase White, David Jaspan, Jay Goldberg

**Affiliations:** Department of Obstetrics and Gynecology, Einstein Medical Center Philadelphia, Philadelphia, Pennsylvania

*To the Editor*—In their recent publication, Goldfarb et al^1^ reported a low prevalence of coronavirus disease 2019 (COVID-19), 1.5%, among asymptomatic pregnant women in Boston presenting for admission to labor and delivery between April 18, 2020, and May 5, 2020. Noting that their rate was substantially lower than that reported in New York City, the authors theorized that it might be due to their patients (1) being tested >30 days after physical distancing orders were in place; (2) the population density of Boston being less than New York City; and (3) New York women underreporting symptoms due to New York hospitals banning support people from labor and delivery.^1^

Studying similar universal screening in pregnant women presenting to labor and delivery at Einstein Medical Center Philadelphia during the same time frame as the Boston study, we found that 9.6% of 114 consecutive asymptomatic women tested positive for severe acute respiratory coronavirus virus 2 (SARS-CoV-2). None of those 11 SARS-CoV-2–positive pregnant women had any COVID-19–related symptoms.

The much higher rate of asymptomatic COVID-19 infections that we found (9.6% vs 1.5%) cannot be explained by the 3 theories proposed by Goldfarb et al. Our Philadelphia COVID-19 testing data are from the same period as the Boston study, when physical distancing orders were also in place. Although Boston does have fewer people per square mile (13,841) than New York City (27,000), Philadelphia has an even lower population density (11,854 people per square mile). On March 28, 2020, prior to the Boston study’s time frame (and ours), Governor Andrew Cuomo announced an executive order that New York hospitals were required to allow 1 person to accompany a patient throughout their labor and delivery. This was issued several days after 2 major New York City hospital systems banned support people from labor and delivery rooms because of the coronavirus pandemic in effort to protect patients, babies, and labor and delivery healthcare providers.^2^

Based upon our findings, as well as others^3^, the very low rate of asymptomatic pregnant women infected with SARS-CoV-2 in the Boston study may be an outlier during the early stages of the pandemic, with a more accurate infection rate being much higher. The higher rate of asymptomatic pregnant women infected with SARS-CoV-2, who may still infect healthcare providers and others, demonstrates the importance of universal testing of pregnant women admitted to labor and delivery, as well as precautions such as mask wearing and hand washing.
